# CONFORMAL PREDICTION OF RESIDUAL COGNITION IN AGING AND ALZHEIMER’S DISEASE

**DOI:** 10.1093/geroni/igae098.3644

**Published:** 2024-12-31

**Authors:** Ime Essien, Laura McDaniel, Jeremy Walston, David Bennett, Peter Abadir, Rama Chellappa

**Affiliations:** Johns Hopkins University, Baltimore, Maryland, United States; Johns Hopkins University, Baltimore, Maryland, United States; Johns Hopkins University, Baltimore, Maryland, United States; Rush University Medical Center, Chicago, Illinois, United States; Johns Hopkins University, Baltimore, Maryland, United States; Johns Hopkins University, Baltimore, Maryland, United States

## Abstract

Cognitive reserve (CR) and resilience in aging and Alzheimer’s disease (AD) have gained significant attention in recent research. We explore cognitive resilience by examining how individuals maintain cognitive function despite neuropathological burden, particularly in the context of AD in Religious Orders Study and Rush Memory and Aging Project (ROSMAP) cohort. Our approach involves modeling residual cognition, defined as the difference between observed cognitive function and expected function based on neuropathological burden. Conformal prediction is applied to generate prediction intervals for global cognitive scores based on beta-amyloid and tau protein levels and demographic data. This provides a valid measure of uncertainty to derive our residual cognition. This method allows us to identify cases where beta-amyloid and tau protein levels are misaligned with final cognitive diagnoses, providing valuable feature interpretability. The study stratifies participants using the Clinical consensus diagnosis of cognitive status at time of death into control, asymptomatic AD (AsymAD), and AD groups to investigate cognitive maintenance in the presence of AD-associated neuropathological changes. By comparing residuals across different diagnostic groups, we gain insights into factors contributing to cognitive resilience. We use the clinical diagnosis of cognitive status as a validation measure to examine how well our residual cognition measure predicts clinical outcomes. Our research builds upon previous work on CR measurement, including decomposition approaches, the Alzheimer’s Disease Cognitive Resilience Score, and the residual reserve index. The findings will help inform interventions to promote cognitive health in aging populations.

